# A Distinctive Strategy for Catheter Ablation in Patients With Bilateral Common Ostium in the Inferior Pulmonary Veins: A Case Report

**DOI:** 10.1002/ccr3.70042

**Published:** 2024-12-31

**Authors:** Zhaoyang Wei, Linan Gong, Zanzan Wang, Zheng Zuo, Zhiguo Zhang

**Affiliations:** ^1^ Department of Cardiology The First Hospital of Jilin University Changchun Jilin China

**Keywords:** atrial fibrillation, case report, catheter ablation, common ostium

## Abstract

Pulmonary vein anatomical variations are frequently observed in atrial fibrillation patients undergoing catheter ablation. However, when it comes to patients with atrial fibrillation and bilateral common ostium in the inferior pulmonary veins, using a bilateral circumferential pulmonary vein isolation approach during catheter ablation heightens the risk of esophageal injury. At present, there is no established standard catheter ablation strategy for such cases. A 47‐year‐old female with atrial fibrillation underwent catheter ablation. Prior to the procedure, a left atrial computed tomography angiography indicated a common ostium of the left and right inferior pulmonary veins. During mapping, a low‐voltage area was found in the patient's left atrial posterior wall. To avoid esophageal injury and effectively isolate both pulmonary veins and the low‐voltage area with minimal ablation points, we used a single‐ring ablation approach. In a 12 month follow‐up, the patient had no atrial fibrillation recurrence.

## Graphical abstract

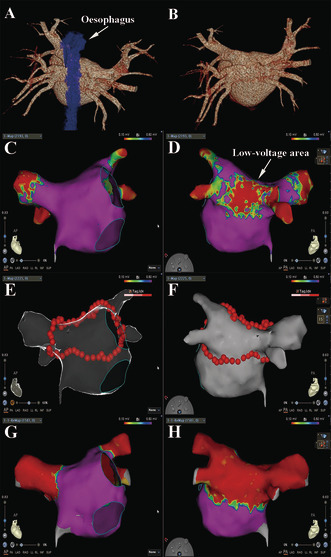

**Summary**

- Circumferential pulmonary vein isolation has become the standard method for radiofrequency ablation of atrial fibrillation.
- In patients with atrial fibrillation and bilateral pulmonary vein common ostium, conventional circumferential pulmonary vein isolation increases the risk of esophageal injury.
- The application of a single‐ring ablation technique minimizes the risk of esophageal injury.

## 1 Introduction

In patients with atrial fibrillation (AF) who undergo catheter ablation therapy, isolating the pulmonary veins is essential. Anomalies in the pulmonary veins' anatomy can greatly impact the procedure's success. Notably, 23%–38% of AF patients undergoing catheter ablation show variations in pulmonary vein anatomy [1]. When there is a common ostium of bilateral inferior pulmonary veins, it complicates vein ablation and increases the risk of esophageal injury. Therefore, safer and more effective ablation strategies are needed. In our experience, we encountered a case with this anomaly and adopted an unconventional ablation approach, resulting in favorable outcomes.

## 2 Case History/Examination

**FIGURE 1:**
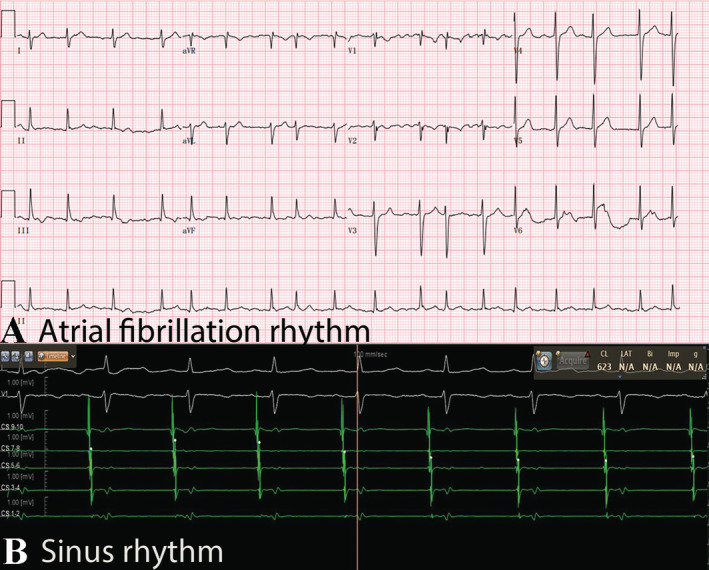
Preoperative surface electrocardiogram (A) and intracardiac electrocardiogram of the patient (B).

**FIGURE 2:**
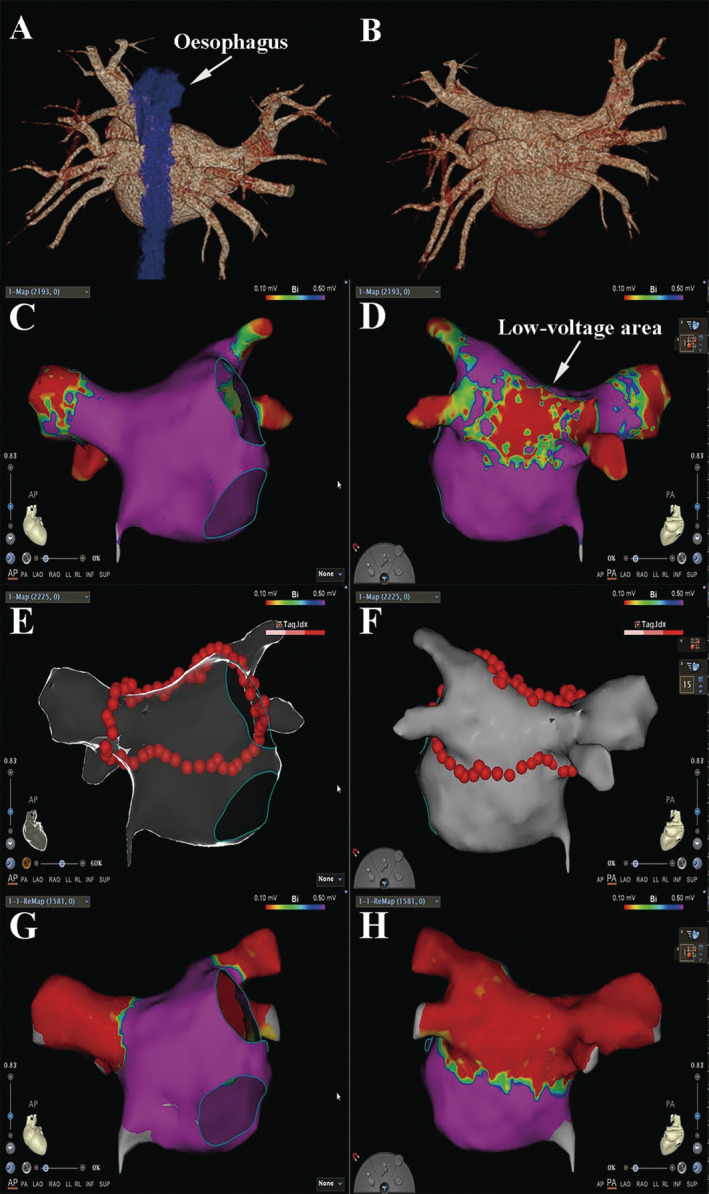
Preoperative left atrial computed tomography angiography revealed a common ostium of the bilateral inferior pulmonary veins into the left atrium, with the common ostium being close to the esophagus (A, B). Intraoperatively, mapping confirmed predominantly uniform high‐voltage in most areas of the left atrium (C), with only the posterior wall exhibiting a low‐voltage area (D). During catheter ablation, a single‐ring ablation strategy was employed (E: Anterior–posterior position, F: Posterior–anterior position). Post‐catheter ablation, successful isolation of both pulmonary veins and the low‐voltage area in the left atrial posterior wall was achieved (G: Anterior–posterior position, H: Posterior–anterior position).

A 47‐year‐old female patient came to our hospital reporting 2 months of palpitations and chest discomfort, with an electrocardiogram confirming paroxysmal AF. Despite treatment with rivaroxaban and metoprolol, her palpitations did not relieve significantly, leading her to seek care at our hospital. Upon admission, the patient's physical examination revealed AF. Routine blood tests showed no abnormalities, while an electrocardiogram confirmed the presence of AF (Figure 1A). The echocardiogram indicated an estimated left atrial volume index of 30 mL/m^2^ and an ejection fraction of 63%. Further assessment via transoesophageal echocardiography revealed no thrombus in both the left atrium and left atrial appendage. Additionally, we conducted preoperative left atrial computed tomography angiography to obtain early insights into the left atrial structure. The results revealed a common ostium of the left and right inferior pulmonary veins. (Figure 2A,B). After confirming the diagnosis of AF, the decision was made to proceed with catheter ablation for the patient.

## 3 Methods

Following the patient's admission to the catheterization room, standard preoperative preparations, interatrial septal puncture, and catheter placement were carried out. At that time, the patient's intracavitary electrocardiogram indicated sinus rhythm (Figure 1B). Guided by the CARTO system (CARTO 3, Biosense Webster, US), mapping of both the left atrium and bilateral pulmonary veins was successfully performed using the PENTARAY catheter (Biosense Webster). During this process, a low‐voltage area was identified in the posterior left atrium, and it was observed that the left and right inferior pulmonary veins shared a common ostium (Figure 2C,D). Given the patient's unique atrial anatomy and the presence of the low‐voltage area, a decision was made to proceed with a single‐ring ablation approach (Figure 2E,F). In this case, we employed ablation index‐guided high‐power ablation to determine the energy for each ablation point. The power was set to 45 watts. The ablation index values were 480 at the roof of the left atrium, 450 at the floor of the left atrium, and 400 near the esophagus at the floor of the left atrium. A total of 64 ablation points were used to complete the entire ablation ring. The single‐ring ablation successfully isolated all four pulmonary veins and automatically addressed the low‐voltage area in the posterior left atrium without the need for additional ablation (Figure 2G,H). The ablation trajectory starts from the right superior pulmonary vein, proceeds through the top of the left atrium, left pulmonary veins, and the bottom of the left atrium, and finally connects back to the starting point of ablation. Double potentials can be identified in the right inferior pulmonary vein during the ablation process. The ablation catheter used is the CARTO Smart Touch ablation catheter (Biosense Webster), with an ablation energy of 45 watts and an ablation index of 450. The patient's vital signs remained stable throughout and after the procedure, allowing for her safe return to her ward.

## 4 Outcome and Follow‐Up

The patient was discharged 2 days after the procedure. 12 months postoperatively, the patient did not experience a recurrence of palpitations, and the follow‐up electrocardiogram still showed sinus rhythm.

## 5 Discussion

### 5.1 Anomalous Pulmonary Vein Anatomy and AF

Research indicates the presence of left atrial tissue within the pulmonary veins, where pacemaker cells trigger AF [2, 3]. Consequently, the isolation of pulmonary veins constitutes a foundational procedure in AF surgery. However, abnormalities in pulmonary vein anatomy frequently pose challenges in ablation procedures, diminishing treatment efficacy. In embryonic development, pulmonary veins gradually differentiate into four branches [4]. Deviations in this developmental process can lead to atypical pulmonary vein anatomy, such as the infrequent occurrence of a common ostium in bilateral inferior pulmonary veins [5].

### 5.2 Common Ostium of Bilateral Inferior Pulmonary Veins and Low‐Voltage Area in the Left Atrium Posterior Wall

A thorough understanding of left atrial anatomy is crucial for the success of catheter ablation. Preoperative left atrial computed tomography angiography imaging provides the following information: (a) Detailed anatomical structure of the left atrium, including variations in the anatomy of the left atrium and pulmonary veins; (b) Exclusion of left atrial thrombus; (c) Determination of left atrial volume [6]. Studies have highlighted the common ostium in bilateral inferior pulmonary veins as a crucial focal point for paroxysmal AF, necessitating its ablation [5, 7, 8]. Additionally, low‐voltage areas within the left atrium may act as triggers for AF, aside from the pulmonary veins [9]. This could be associated with the common embryonic origin shared by the pulmonary veins and the left atrial posterior wall [4]. In this case and in previous reports, patients with common ostium in the bilateral inferior pulmonary veins and low‐voltage areas in the left atrial posterior wall were observed [8, 10]. Studies have indicated that the development of this low‐voltage area could be associated with the early termination of pulmonary vein development and the presence of atrial fibrosis [4, 9]. Prior studies have emphasized the necessity of isolating the left atrial posterior wall, even in the absence of measurable potentials, to prevent the recurrence of AF [8, 11]. Therefore, isolating the left atrial posterior wall region is necessary, but this will increase the number of ablation points.

### 5.3 Choice of Ablation Strategy

In this case, preoperative imaging suggested a common ostium for the bilateral inferior pulmonary veins. During the procedure, mapping indicated a low‐voltage area on the posterior wall of the left atrium. Therefore, the single‐ring ablation strategy was chosen. Studies have indicated that the long‐term prognosis of AF treated with the single‐ring ablation strategy is comparable to conventional encircling pulmonary vein isolation strategies and can isolate a larger left atrial area [12]. In this case, the patient had bilateral common ostium in the inferior pulmonary veins. Additionally, a natural low‐voltage area was identified in the left atrial posterior wall, even though it lacked measurable electrical potentials. In previous case reports, for atrial fibrillation patients with a common ostium of the bilateral lower pulmonary veins, Li et al. [13] used second‐generation cryoballoon ablation, while Yu et al. [5] employed the tri‐circle strategy. These methods inevitably increased the ablation dose on the posterior wall of the left atrium. We employed the single‐ring ablation strategy, resulting in the isolation of both pulmonary veins and the entire left atrial posterior wall. Notably, this strategy reduced the number of required ablation points and eliminated the need for ablation in the left atrial posterior wall, thus reducing the risk of esophageal injury.

The main distinction between the single‐ring ablation strategy and traditional ablation techniques lies in the ablation route, which leads to differences in outcomes and complications. Specifically, the single‐ring strategy avoids the area of the left atrial posterior wall near the esophagus, thereby effectively preventing esophageal injury [14]. Additionally, while isolating the pulmonary veins, the single‐ring strategy also achieves electrical isolation of the left atrial posterior wall, potentially reducing the recurrence rate of atrial fibrillation [8, 9].

Although the application of the single‐ring ablation strategy itself lowers the risk of operational complications, some challenges remain. Notably, the single‐ring ablation line is relatively long, making it difficult to isolate the pulmonary veins during initial ablation attempts. This requires an experienced operator to ensure a high success rate [15]. Furthermore, positioning the ablation ring of a single‐ring strategy too anteriorly may damage the Bachmann's bundle, leading to an atrial conduction block and potentially increasing the risk of postoperative atrial flutter or fibrillation. Lastly, current pulsed field ablation technology allows for pulmonary vein isolation without damaging adjacent tissues, making it a viable option for patients with complex anatomical structures. However, its application is not yet widespread, and its long‐term prognosis requires further research [16].

### 5.4 The Significance of This Case Report

Patients with bilateral common ostium in the inferior pulmonary veins may have low‐voltage areas in the left atrium, necessitating isolation even without detectable electrical potentials. Standard circumferential pulmonary vein ablation in these patients raises the risk of atrio‐esophageal fistula formation. Using the single‐ring ablation strategy may provide an effective treatment option with lower risks. In addition, relying solely on mapping systems for left atrial anatomy may not adequately identify common ostium. Therefore, comprehensive preoperative imaging is crucial [17].

## 6 Conclusion

For patients with AF and bilateral common ostium of the inferior pulmonary veins, isolating the low‐voltage area in the left atrial posterior wall is crucial for preventing AF recurrence. However, conventional ablation strategies pose a risk of esophageal injury. The use of single‐ring ablation can simultaneously isolate the pulmonary veins and the left atrial posterior wall while minimizing the risk of esophageal injury.

## Author Contributions

**Zhaoyang Wei:** writing – original draft, writing – review and editing. **Linan Gong:** supervision, writing – review and editing. **Zanzan Wang:** supervision, writing – review and editing. **Zheng Zuo:** supervision, writing – review and editing. **Zhiguo Zhang:** conceptualization, supervision, writing – review and editing.

## Consent

The authors confirm that written consent for submission and publication of this case report, including images and associated text, was obtained from the patient in line with COPE guidance.

## Conflicts of Interest

The authors declare no conflicts of interest.

## Data Availability

The data underlying this article will be shared upon reasonable request to the corresponding author.
